# Cooling and physiology during parent cuddling infants with neonatal encephalopathy in usual care: CoolCuddle‐2 study

**DOI:** 10.1111/apa.17466

**Published:** 2024-10-25

**Authors:** Ela Chakkarapani, Jenny Ingram, Stephanie Stocks, Lucy Beasant, David Odd, CoolCuddle‐2 study research team

**Affiliations:** ^1^ Centre for Academic Child Health, Bristol Medical School University of Bristol Bristol UK; ^2^ St Michael's Hospital University Hospitals Bristol and Weston NHS Foundation Trust Bristol UK; ^3^ School of Medicine Cardiff University and University Hospital of Wales Cardiff UK

**Keywords:** cuddle, intensive care, neonatal encephalopathy, therapeutic hypothermia

## Abstract

**Aim:**

CoolCuddle, enabling parents to cuddle their babies with neonatal encephalopathy (NE) during therapeutic hypothermia and intensive care (TH), was developed in research settings. To determine the impact of implementing CoolCuddle in usual care in six diverse neonatal intensive care units on the cooling process and intensive care.

**Methods:**

This vital sign cohort study embedded within the CoolCuddle implementation study enrolled 36 infants receiving TH for NE. Nurses received training on CoolCuddle and a standard operating procedure using an instruction video. After consenting, parents experienced up to 2 h of CoolCuddle with 30 min of pre‐ and post‐cuddle observation. We used multilevel, clustered linear modelling to assess the physiological stability in temperature, cardio‐respiratory and neurophysiology across the CoolCuddle.

**Results:**

In 60 CoolCuddles over 93.12 h, respiratory parameters, heart rate or neurological function did not vary between the epochs (*p* > 0.05). During cuddle, sleep–wake cycling on amplitude‐integrated EEG increased (*p* = 0.008) and there was weak evidence of lower pain scores (*p* = 0.08). No adverse effects were observed.

**Conclusion:**

Implementing CoolCuddle with support in usual practice maintained physiological stability and did not significantly affect the cooling process or intensive care, and may improve infant comfort. Ongoing monitoring of adverse effects when implementing CoolCuddle is recommended.

**Abbreviations**
aEEG: amplitude‐integrated electroencephalogram
BP: blood pressure
EEG: electroencephalogram
End‐tidal: CO_2_ end‐tidal carbon dioxide
NE: neonatal encephalopathy
NICUs: neonatal intensive care units
NoMAD: Normalisation MeAsure Development
N‐PASS: neonatal pain agitation and sedation scale
SaO_2_
: peripheral oxygen saturation
TH: therapeutic hypothermia

**Key notes**

- Whether parents cuddling their babies during therapeutic hypothermia (CoolCuddle) during usual care impacted cooling therapy or intensive care was unknown.
- CoolCuddle implemented in usual care maintained physiological stability, improved infant's comfort without impacting the cooling therapy or intensive care or leading to adverse effects.
- CoolCuddle can be implemented in usual practice using the animation videos we produced for staff and parents with ongoing monitoring for adverse effects.

## 1 INTRODUCTION

Therapeutic hypothermia with intensive care (TH) is the standard care for infants with neonatal encephalopathy after birth asphyxia (NE) in high‐income settings.^1^ TH is typically administered using a servo‐controlled whole body cooling device with a wrap or blanket that envelops the body and limbs of the infant.^2^ It is usual clinical practice to not disturb the infants during TH and parents usually are not allowed to cuddle their babies during TH due to concerns of impacting the cooling process or intensive care.^3^ Parents reported that this physical and psychological separation from their babies during TH impacted their ability to bond with their baby and initiate breastfeeding.^4^, ^5^, ^6^ It is well known that healthy parent–infant bonding and breastfeeding impact cognitive development of children.^7^, ^8^ Given that the children cooled for NE have difficulties with cognitive skills at early school age,^9^, ^10^, ^11^ it is even more important to enhance the parent–infant bonding and breastfeeding for infants with NE. Additionally, brain connectivity is disrupted in children cooled for NE at school age^12^, ^13^ and facilitating early parent–infant interactions during TH might improve the brain connectivity.^14^ Therefore, we developed a nurse‐led intervention called CoolCuddle that enables parents to cuddle their babies safely for up to 2 h during TH and intensive care. We showed that when CoolCuddle was delivered in a controlled environment under the supervision of a research advanced neonatal nurse practitioner in two level 3 neonatal intensive care units (NICUs), there were no adverse effects, which were defined *apriori*, including dislodgement of the endotracheal tube, vascular catheters, urinary catheters or EEG electrodes resulting in needle stick injury. Additionally, there was no clinically significant effect on the rectal temperature and cardiorespiratory parameters (CoolCuddle‐1 study).^15^ Although the changes in rectal temperature and cardiorespiratory parameters were not clinically significant, there were measurable increases in rectal temperature and bandwidth of the amplitude‐integrated electroencephalogram (aEEG) and decrease in oxygen saturation during the cuddle as well as an increase in the end‐tidal CO_2_ and mean arterial blood pressure after the cuddle, compared with the pre‐cuddle epoch. It is not known whether these changes in temperature and intensive care measures would be replicated and whether adverse effects including dislodgement of endotracheal tubes, vascular or urinary catheters or EEG leads would occur, when CoolCuddle is implemented in routine usual care in diverse level 3 neonatal intensive care settings.

In the CoolCuddle‐2 study, we rolled out the CoolCuddle in six level 3 NICUs across England with a nurse champion training a small group of nurses with a video produced by the study investigators and an adaptable, rigorous standard operating procedure.^16^ The nurses looking after the baby then supported the parents with the CoolCuddle during usual care. We aimed to determine whether CoolCuddle implemented in usual care in diverse level 3 neonatal intensive care settings will impact the cooling process or intensive care or safety profile.

## 2 METHODS

### 2.1 Study design

This was a vital sign cohort study, nested within the CoolCuddle implementation study, conducted in six level 3 NICUs across England between September 2022 and August 2023. The NICUs included in the study were University Hospital Southampton, Birmingham Women's Hospital, Manchester University Hospital, Nottingham University Hospital, University Hospitals of Leicester and South Tees Hospitals NHS Trust. The study initially commenced at five sites and South Tees was added to the study in February 2023 to boost recruitment. CoolCuddle2 received ethics approval from North West‐Greater Manchester West Research Ethics Committee (22/NW/0141) and HRA approval in June 2022.

### 2.2 Participants

Parents and their infants born at or above 35 weeks gestation undergoing TH using a servo‐controlled cooling machine and intensive care for NE were eligible for the study. Parents of babies who are unable to complete the consent form, postnatal depression and parent–infant bonding questionnaires due to lack of proficiency in English; or parents who were under the age of 16 were excluded. Babies who were receiving considerable levels of intensive care including high‐frequency oscillation or receiving mean airway pressure >15 cm H20 or receiving oxygen >70% or having a chest drain or receiving three or more inotropes or in status epilepticus were excluded as indicated in the study protocol.^17^

### 2.3 Study procedures

Once a core group of nurses were trained, they explained the process involved with the CoolCuddle to the parents and obtained verbal consent, which was documented in the clinical notes. Parents were comfortably seated on a chair close to their baby with a pillow on their lap. The nurse looking after the baby prepared the baby for the cuddle by grouping and aligning the cables and infusion lines using a Velcro strap, covering the baby with a sheet, and then moved the baby with the help of one or two nurses over to the pillow on the parents' lap. CoolCuddle lasted for up to 2 h. Routine intensive care monitoring, including single‐channel amplitude integrated electroencephalogram (aEEG) monitoring, continued during the cuddle.

### 2.4 Data collection

We recorded routinely measured data including core and surface temperature, cardio‐respiratory and neurophysiological data before the cuddle, for every 30 min during the cuddle, and again once the infant was settled back in the intensive care space. Data were collected on the case report form, which was part of the standard operating procedure checklist used to administer each CoolCuddle. Not all measures (especially invasive blood sampling) were performed in all infants at all time points. Cardio‐respiratory data included heart rate, mean blood pressure (BP), ventilatory parameters, peripheral oxygen saturation (SaO_2_) and blood gases. Analgesic and inotropic support doses were collected. Pain was scored during the pre‐cuddle, cuddle and post‐cuddle using Neonatal Pain Agitation and Sedation Scale (N‐PASS).^18^ We collected data on adverse events including accidental extubation, dislodgement of vascular or urinary catheters or EEG electrodes and any incidence of needle‐stick injury from EEG electrodes. The research team obtained written consent from parents with legal responsibility after the CoolCuddles were completed before the infant was discharged from the NICU to use the data collected during the CoolCuddle. We used this two‐step consenting process, as CoolCuddle was shown to be relatively safe in the CoolCuddle‐1 study and was being implemented as standard care in the usual practice in this study and therefore does not need written consent. However, to use the routinely collected data from the babies for research we needed to get written consent. None of the parents who were approached for written consent after the CoolCuddle refused to consent to use routinely collected data.

Parents participated in reporting postnatal depression and parent–infant bonding at 5–7 days and 8 weeks postpartum, and these data are reported elsewhere.

### 2.5 Outcomes

CoolCuddle‐2 study was a prospective cohort study using an implementation study design employing the intervention developed in CoolCuddle‐1. The primary aim of this work was to investigate physiological stability across the CoolCuddle period, by describing the changes seen in physiological measures before, during and after the CoolCuddle, and if specific measures increased, or decreased, when compared to the pre‐cuddle period; using the same methodology as reported in our previous work.^15^ The primary outcome was physiological stability, defined as degree of variation across the three measures including core and surface temperature, cardiorespiratory and neurophysiology parameters. The individual components of the primary outcome included changes in the core and surface temperature, cardiorespiratory and neurophysiology parameters.

The sample size was chosen to provide adequate precision for the process evaluation aspect of implementing CoolCuddle across the six NICUs using the Normalisation MeAsure Development (NoMAD) assessment.^17^ The CoolCuddle‐2 project was designed as an implementation science project, and the number of units, and the likely recruitment in each, defined the size of the study. The data describing the qualitative, implementation and parent mood and bonding components of the study including the NoMAD assessment were part of the second work package of the project which has been submitted to another peer‐review journal.

### 2.6 Statistical analysis

Analysis was based on our previous work.^15^ Initially, the demographic characteristics of the enrolled infants were derived. Data were collapsed down to the three epochs (pre, during and post‐cuddle), using means for continuous data and the highest/worst measure for the categorical measures (i.e. any seizure, worst EEG grading, absence of sleep–wake cycling and any pain score >0) in that period. Next, a multilevel, clustered linear model for the continuous measures (with the infant being the highest level, and then cuddle) was derived; with the likelihood ratio test used to assess if there was evidence of a difference between the three periods (the primary analysis); and absolute difference in measures (with 95% confidence intervals) compared to the pre‐cuddle period derived. A logistic model with the same structure was then derived for binary measures (sleep–wake cycling, a high aEEG score). Data were not imputed, so analysis was restricted to where at least one measure was present within each epoch, and so denominators vary. Results are presented as arithmetic mean (SD), mean change (95% CI), number (%) or Odds Ratio (95% CI) as appropriate. Analysis was performed in Stata 17.

## 3 RESULTS

**FIGURE 1:**
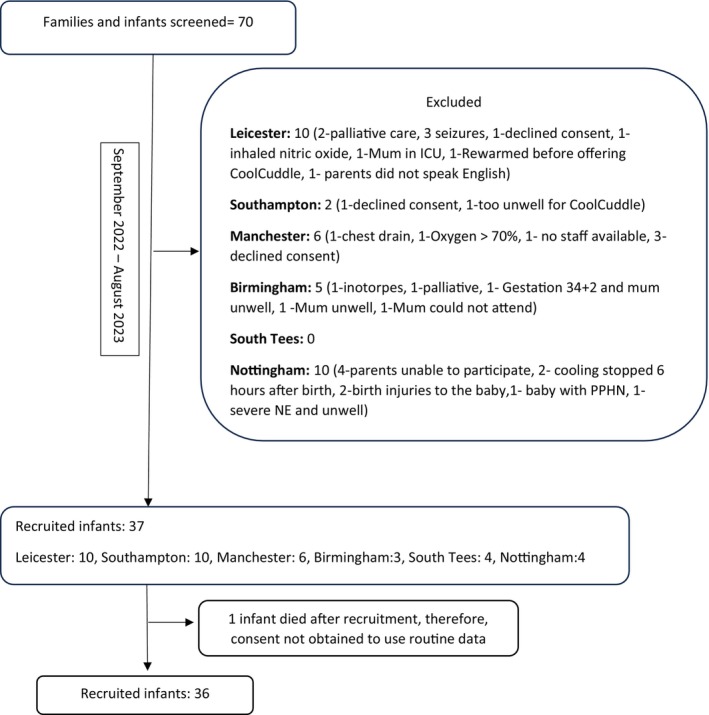
Flow chart showing study recruitment.

Between September 2022 and August 2023, the research teams across the six NICUs screened a total of 70 families and infants (Figure 1). Thirty‐three families and infants were excluded due to fulfilling one of the exclusion criteria (redirection of care: 4; birth injuries to back or shoulder: 2; exclusion criteria: seizures 3, too unwell: 2; inhaled nitric oxide: 2, chest drain: 1, high levels of oxygen need: 1, inotropic support: 1, preterm infant: 1, finished cooling or passively cooled until 6 h after birth: 2, parent did not speak English: 1; parent unwell or unable to participate: 7; declined consent: 5; staff unavailable: 1). Thirty‐seven families were recruited, of whom, one infant died after recruitment and therefore consent was not obtained to use the routinely collected data.

In total, 36 infants were enrolled in the study, across 6 units; with Southampton (*n* = 10) and Leicester (*n* = 9) recruiting over half of them (Table 1). The mean age of the mothers was 29.4 (SD 8.3) years, and most (89%) were of a white background. Fathers were slightly older (31.0 (SD 9.4)) and of similar ethnicity. Nearly half of the mothers were primiparous (47%). Further parental details are shown in Table 1. The median gestation was 38.5 (IQR 37.6–39.7) weeks of gestation, and the mean cord pH was 7.01 (SD 0.16). Eight (25.0%) infants had only a mild grade of NE, while four (12.5%) had a Grade 3. In total, 36 infants underwent 60 CoolCuddles with a mean duration of 1.55 h per infant and a cumulative duration of 93.12 h of cuddling. Most first CoolCuddles occurred on the second (*n* = 12 (33.3%)) or third (*n* = 13 (36.1%)) calendar day of life. Of the 36 infants, 14 (39%) received invasive ventilation. During 37 CoolCuddles, morphine was infused for analgesia, with a mean dose of 10.6 mcg/kg/h. Dobutamine was used during two CoolCuddles, at a mean dose of 8.8 mcg/kg/min, while Dopamine was used in 1 (at 7.5 mcg/kg/min). Across the 60 cuddles, not all physiological measures were recorded for all infants (especially blood gas measures which were done at the discretion of the attending clinical team) (Table 2).

**TABLE 1:** Characteristics of the study population.

Measure	*N* ^a^	Total
Neonatal unit		
Birmingham	3	–
Leicester	9	–
Manchester	6	–
Nottingham	4	–
South tees	4	–
Southampton	10	–
Maternal characteristics		
Age (years) (mean (SD))	36	29.4 (8.4)
Race – White	36	32 (89%)
Pregnancy characteristics		
Primiparous	36	17 (47%)
Induction of labour	35	13 (37%)
Pregnancy complications	36	13 (36%)
Intrapartum complications	36	30 (83%)
Lower segment caesarean section (LSCS)	36	21 (58%)
Breech	34	4 (12%)
Pyrexia >38°C, *n* (%)	34	1 (3%)
Paternal characteristics		
Age (years) (mean (SD))	26	31.0 (9.4)
Race – White	31	29 (94%)
Infant characteristics		
Sex (male)	36	21 (58%)
Gestation weeks (median (IQR))	36	38.5 (37.6–39.7)
Birth weight g, mean (SD)	36	3280 (650)
Head circumference cm, mean (SD)	23	33.5 (33.0–35.0)
Transferred from LNU or SCBU for cooling	36	17 (47%)
Cord blood gas		
pH, mean (SD)	34	7.01 (0.16)
Base excess (SD)	34	−11.8 (10.1)
Lactate (SD)	30	11.1 (5.4)
Apgar scores		
1 min (median (IQR))	30	2 (1–3)
5 min (median (IQR))	32	4 (2–6)
Need for respiratory support >10 min	36	36 (89%)
NE grade	32	
I		8 (25.0%)
II		20 (62.5%)
III		4 (12.5%)
aEEG abnormality before TH^a^	29	
Normal		6 (21%)
Moderately abnormal		19 (65%)
Severely abnormal		4 (14%)
Cardiac compressions delivered	36	12 (33%)
Resuscitation drugs given	36	10 (28%)
Age when reached (hours) 33·5°C (mean (SD))	36	5.0 (4.0–6.3)

*Note*: Percentages were rounded up or down for easy interpretation.

^a^ Moderately abnormal aEEG: minimum amplitude of aEEG ≤5 microvolts and maximum amplitude of aEEG >10 microvolts; severely abnormal aEEG: minimum amplitude of aEEG <5 microvolts and maximum amplitude <10 microvolts.

**TABLE 2:** Summary Values of the respiratory, cardiovascular haemodynamics and core temperature data.

Variable	*N* ^a^	Pre‐cuddle	*N* ^a^	During cuddle	*N* ^a^	Post‐cuddle	*p* Value^b^
Respiratory parameters							
PIP cmH_2_0	22	17.0 (6.1)	27	16.0 (5.6)	22	17.2 (6.5)	0.2293
PEEP cmH_2_0	22	5.3 (0.7)	27	5.4 (0.7)	22	5.1 (0.8)	0.2035
MAP cmH_2_0	23	8.5 (4.1)	27	7.8 (1.8)	22	7.7 (2.0)	0.4571
FiO_2_%	48	22.9 (4.7)	51	23.1 (4.9)	49	22.5 (4.6)	0.301
SaO_2_%	60	98.1 (2.7)	60	98.0 (1.5)	59	98.3 (2.1)	0.4974
T_I_ seconds	20	0.38 (0.4)	25	0.38 (0.04)	21	0.38 (0.04)	0.8418
Tidal volume mL	21	16.2 (7.0)	25	16.3 (4.9)	21	16.7 (5.1)	0.7995
Respiratory rate	59	42.1 (11.5)	60	42.7 (9.4)	59	43.5 (12.0)	0.5682
Blood gas measures							
pH	31	7.35 (0.05)	2	7.37 (0.04)	26	7.34 (0.05)	0.8104
pO_2_ kPa	31	10.6 (3.5)	2	11.3 (4.9)	26	8.5 (2.9)	0.0163
pCO_2_ kPa	31	5.8 (1.2)	2	4.9 (0.0)	26	6.0 (1.1)	0.8336
Glucose mmol/L	31	4.5 (1.7)	2	4.5 (0.1)	26	4.5 (1.4)	0.9240
Lactate mmol/L	30	1.7 (1.2)	2	1.3 (0.1)	25	1.8 (1.3)	0.7226
Cardiovascular							
Mean BP mmHg	60	53.3 (10.1)	58	50.5 (10.2)	60	51.6 (10.1)	0.0457
Heart rate beats/min	60	105 (16.1)	60	105 (13)	59	106 (14.7)	0.6712
Neurology							
Seizures	57	2 (3.5%)	59	1 (1.7%)	53	0 (0.0%)	NA
aEEG							
Lower margin (μV)	51	4.9 (1.9)	53	5.0 (1.1)	50	5.1 (2.3)	0.6580
Upper margin (μV)	51	16.5 (9.3)	53	15.7 (8.2)	50	15.6 (9.4)	0.1378
Bandwidth μV	51	11.6 (10.0)	53	10.7 (8.6)	50	10.5 (9.6)	0.1136
Sleep–Wake cycling	52	27 (51.9%)	52	32 (61.5%)	46	25 (54.4%)	0.0085
Pain score >0	28	11 (39.3%)	28	6 (21.4%)	26	8 (30.8%)	0.0865
Temperature							
Peripheral temp°C	59	32.8 (1.2)	58	32.1 (0.8)	58	32.7 (1.3)	0.3106
Rectal temp°C	60	33.6 (0.6)	60	33.6 (0.5)	59	33.6 (0.5)	0.9526

Abbreviations: FiO_2_, fraction of inspired oxygen; MAP, mean airway pressure; NA, not available; pCO_2_, partial pressure of carbon dioxide; PEEP, peak end expiratory pressure; PIP, peak inspiratory pressure; pO_2_, partial pressure of oxygen; SaO_2_, peripheral oxygen saturation; T_I_, inspiratory time.

^a^ Number of observations for that measure, in that epoch.

^b^ Probability of a difference in measures between epochs.

The primary outcome of physiological stability was not impacted during CoolCuddle. The components of the primary outcome were not affected either. There was little evidence that any respiratory parameters varied over the 3 time periods (all *p* > 0.05), and while pO_2_ changed across the study period (*p* = 0.0163), there was little to suggest individually different levels during (mean difference −0.36 (−3.03 to 2.32)) or after the cuddle (mean difference −1.32 (−2.18 to 0.46)) than before (Table 2 and 3). While there was little evidence of a difference in heart rate (*p* = 0.6712), mean BP varied across the three periods (*p* = 0.0457), but again, like pO_2_ there was little to suggest individual differences during (mean difference −2.75 (−4.93 to 0.57)) or after the cuddle (mean difference −1.73 (−3.89 to 0.42)) compared with pre‐cuddle. Equally, while most measures of neurological function were similar across the measures, there was evidence of a difference in sleep–wake cycling on amplitude integrated EEG (*p* = 0.0085), and weak evidence of a change in the pain score (*p* = 0.0865) (Table 2 and 3).

**TABLE 3:** Changes in summary values of the respiratory, cardiovascular haemodynamics and core temperature data compared to pre‐cuddle period.

Variable	*N* ^a^	Pre‐cuddle	*N* ^a^	During cuddle	*N* ^a^	Post‐cuddle
Respiratory parameters						
PIP cmH_2_0	22	Ref	27	−0.94 (−2.13 to 0.24)	22	0.26 (−0.95 to 1.48)
PEEP cmH_2_0	22	Ref	27	0.03 (−0.12 to 0.19)	22	−0.14 (−0.30 to 0.03)
MAP cmH_2_0	23	Ref	27	−0.69 (−1.78 to 0.41)	22	−0.51 (−1.66 to 0.64)
FiO_2_%	48	Ref	51	0.00 (−0.54 to 0.55)	49	−0.38 (−0.93 to 0.18)
SaO_2_%	60	Ref	60	−0.07 (−0.61 to 0.46)	59	0.24 (−0.30 to 0.78)
T_I_ seconds	20	Ref	25	0.00 (−0.00 to 0.00)	21	0.00 (−0.00 to 0.00)
Tidal volume mL	21	Ref	25	−0.03 (−1.94 to 1.88)	21	0.56 (−1.41 to 2.54)
Respiratory rate	59	Ref	60	0.50 (−2.02 to 3.01)	59	1.36 (−1.17 to 3.88)
Blood gas measures						
pH	31	Ref	2	0.02 (−0.04 to 0.07)	26	−0.00 (−0.02 to 0.02)
pO_2_ kPa	31	Ref	2	−0.36 (−3.03 to 2.32)	26	−1.32 (−2.18 to 0.46)
pCO_2_ kPa	31	Ref	2	−0.31 (−1.36 to 0.75)	26	0.13 (−0.22 to 0.47)
Glucose mmol/L	31	Ref	2	0.17 (−1.27 to 1.60)	26	−0.23 (−0.69 to 0.24)
Lactate mmol/L	30	Ref	2	−0.03 (−0.57 to 0.52)	25	−0.07 (−0.26 to 0.11)
Cardiovascular						
Mean BP mmHg	60	Ref	58	−2.75 (−4.93 to −0.57)	60	−1.73 (−3.89 to 0.42)
Heart Rate bpm	60	Ref	60	−0.04 (−3.01 to 2.93)	59	1.16 (−1.83 to 4.15)
Neurology						
Seizures (OR)	57	Ref	59	0.03 (0.00 to 144.92)	53	NA
aEEG						
Lower margin (μV)	51	Ref	53	0.03 (−0.36 to 0.43)	50	0.18 (−0.22 0.58)
Upper margin (μV)	51	Ref	53	−0.77 (−1.78 to 0.24)	50	−1.00 (−2.02 to 0.03)
Bandwidth (μV)	51	Ref	53	−0.81 (−1.92 to 0.30)	50	−1.17 (−2.29 to −0.05)
Sleep–Wake cycling (OR)	52	Ref	52	35.76 (1.01 to 1263.80)	46	2.54 (0.16 to 40.44)
Pain score >0 (OR)	28	Ref	28	0.06 (0.00 to 1.73)	26	0.33 (0.03 to 3.69)
Temperature						
Peripheral Temp°C	59	Ref	58	0.07 (−0.14 to 0.28)	58	−0.10 (−0.31 to 0.11)
Rectal Temp°C	60	Ref	60	−0.03 (−0.13 to 0.07)	59	−0.02 (−0.12 to 0.08)

Abbreviations: FiO_2_, fraction of inspired oxygen; MAP, mean airway pressure; NA, not available; pCO_2_, partial pressure of carbon dioxide; PEEP, peak end expiratory pressure; PIP, peak inspiratory pressure; pO_2_, partial pressure of oxygen; SaO_2_, peripheral oxygen saturation; T_I_, inspiratory time.

^a^ Number of observations for that measure, in that epoch.

When looking at changes during and after the cuddle, compared with the pre‐cuddle period, no respiratory parameter appeared to be different (Table 3); pO_2_ and mean BP measures are discussed above. There were higher odds of sleep–wake cycling being reported during the cuddle than before (OR 35.76 (95% CI 1.01–1263.80)), which did not persist in the post‐cuddle period (OR 2.54 (0.16–40.44)).

There was no evidence of an overall change in the pattern, or of specific measures, of peripheral, or rectal temperatures over the study period. There were no adverse events reported during the study period.

## 4 DISCUSSION

Implementing CoolCuddle in usual care across six tertiary NICUs did not impact the cooling process or intensive care. CoolCuddle involves parents cuddling their babies, while undergoing therapeutic hypothermia for neonatal encephalopathy and intensive care and supported by nurses who had received training on CoolCuddle with an instruction video and a standard operating procedure. There was a measurable increase in the proportion of infants with sleep–wake cycling on their amplitude integrated EEG during the cuddle. There was no impact on the rectal and surface temperature during and after the CoolCuddle. There was weak evidence that the proportion of infants with higher pain scores decreased during the cuddle.

CoolCuddle infants maintained physiological stability and we did not observe any clinically significant changes during or after cuddle in the components of the primary outcome including rectal or surface temperature, cardiorespiratory and neurophysiology parameters. There were no predefined adverse effects reported during CoolCuddle. Our results are consistent with the CooolCuddle‐1 study^15^ and another maternal holding study conducted in more stable infants cooled for NE.^19^ Although there was some evidence that single measures of blood pressure and the pO_2_ may have differed between the pre‐cuddle, cuddle and post‐cuddle epochs, there was little to support an increase or decrease in any measure compared to the pre‐cuddle period suggesting a clinically important change. We observed measurable changes in more physiological parameters, that were clinically non‐significant in the CoolCuddle‐1 study than in the CoolCuddle‐2 study, including oxygen saturation, rectal temperature and aEEG bandwidth during the cuddle, and end‐tidal and pCO_2_ and mean BP after the cuddle. This could be due to the higher frequency of data collection, at every minute in the CoolCuddle‐1 study compared to the routine data collection every 30 min in the CoolCuddle‐2 study. The higher number of infants with lower pain scores during the cuddle, although not statistically significant, suggests that the infants were comfortable and less distressed during cuddles. This is consistent with preterm infants showing lower pain scores when they had skin‐to‐skin contact with their parents before, during and after heel prick, although in our study, infants were not exposed to painful procedures, but had ongoing cooling therapy and intensive care.^20^ We also observed a higher number of infants with sleep–wake cycling during cuddling. Similar results were reported in stable preterm infants undergoing skin‐to‐skin contact with their parents, with more mature sleep organisation.^21^ There is some evidence that the qualities of parent–infant interactions may influence the brain functional connectivity measured using EEG. The frontal‐posterior connectivity was related to maternal responsiveness, reciprocity and positive emotional tone during maternal–infant interactions in 6–11‐month‐old infants.^22^ However, given the small number of infants in this work, the background sedation, and the possibility of artefact, the results should be interpreted with caution.

There was no change in the rectal and surface temperature during or after cuddle in CoolCuddle‐2 study and the rectal temperature change that was observed in CoolCuddle‐1 was an average increase of 0.07°C. Importantly, we did not observe any of the pre‐specified adverse effects including detachment of EEG leads or needle stick injury from the EEG leads or dislodgement of endotracheal tube or arterial, venous or urinary catheters. Combining all these data, we can be reasonably confident that it is safe for parents to experience CoolCuddle for up to 2 h with their infants with moderate‐to‐severe NE undergoing therapeutic hypothermia and intensive care with the support of our resources. To maximise the accessibility of CoolCuddle for NICU staff and parents globally, we have produced animation videos to train staff (https://youtu.be/dC7SriN99SA) and to inform parents (https://youtu.be/ZVN83K0xp7g).

We excluded infants who might be unstable and may not tolerate the move, such as infants requiring considerable intensive care with higher levels of cardiorespiratory support or severe encephalopathy with status epilepticus or infants who in the opinion of the clinical team may not be suitable for the CoolCuddle. It will be useful to investigate whether CoolCuddle might be safe in these infants, particularly in infants who were undergoing redirection of care, as in this instance, CoolCuddle might help parents with their bereavement. We did recruit 12.5% of infants with severe encephalopathy, but it is likely they were stable. In our CoolCuddle‐1 study, where we developed CoolCuddle, one third of enrolled infants had severe encephalopathy and another study of parental holding during therapeutic hypothermia did not enrol infants with severe encephalopathy.^19^ On the other hand, 25% of infants recruited to the current CoolCuddle‐2 study were reported to have mild NE indicating the therapeutic creep in offering therapeutic hypothermia to infants with mild NE.^23^ The majority of the participants were recruited from two participating units. The incidence of NE in the United Kingdom is 2.6/1000 live births (95% CI 2.5–2.8)^24^ and is not equally distributed across the units. Therefore, recruitment varied between 29% and 100% across the participating sites accounting for the exclusions and the variable denominator of the available pool of potential participants to recruit. The common reasons for inability to recruit a family to the study were lack of availability of parents to participate in the study relating to mother being too unwell, followed by parents declining to participate in the study, suggesting some parents might be concerned about the safety of the Cuddle, which led us to produce the animation to explain CoolCuddle to future parents.

With regard to parental factors that might influence the adoption of CoolCuddle in routine practice, we observed that 89% of mothers enrolled in our study were White. We currently do not know about the acceptability of CoolCuddle for parents of other ethnicities and we do not have the ethnicity data on parents who declined consent to CoolCuddle. More than half of the mothers who had CoolCuddle had caesarean section, and therefore, it is feasible for mothers to experience CoolCuddle for up to 2 h with adequate pain relief after a caesarean section, as we found in the CoolCuddle‐1 study.^25^ Indeed, the mean duration of each cuddle was 1.55 h (making a total of 93.12 h of cuddling), suggesting that experiencing CoolCuddle for up to 2 h is acceptable to parents. Nearly half of the infants recruited to the CoolCuddle‐2 study were transferred from the local neonatal units and special care baby units to the level 3 NICUs. This could have impacted parents visiting the cooling centre on the day of birth and, therefore, could have influenced the age when the first Coolcuddle was offered, with 33.3% and 36.1% CoolCuddles occurring on day 2 and day 3 of cooling therapy. We observed a similar pattern in the CoolCuddle‐1 study, where being born in hospitals outside cooling centres delayed parents reaching the cooling centres and coupled with the process of informed consent, contributed to an average age of 50 h after birth when the first Coolcuddle was offered.^15^

Strengths of our study include investigating the physiological stability of CoolCuddle in diverse NICU settings during usual care. Therefore, data obtained from this study will reflect the real‐world safety data of implementing CoolCuddle. Although we recruited only 36 parents and infants to this study, combining CoolCuddle‐1 and CoolCuddle‐2, we have studied 130 CoolCuddle episodes over a cumulative duration of 208 h. Further, the implementation of Coolcuddle needs to be supported with the resources that we have produced, which can be adapted to local settings, to achieve the physiological stability and safety during the CoolCuddle and continue to monitor for any adverse effects. We have not evaluated CoolCuddle in infants who are needing high levels of intensive care and have severe encephalopathy with seizures. Given some of these babies might undergo redirection of care, offering CoolCuddle to these babies will enable parents to bond better with their babies and help them cope with the grief of loss. This was a pragmatic study using routinely collected physiology data. Measuring data more frequently than every 30 min might have shown some changes in the physiological measures, as we observed in CoolCuddle‐1 study; however, they are likely to be clinically non‐significant as we had noted in the CoolCuddle‐1 study.

## 5 CONCLUSIONS

Extending the CoolCuddle process to an additional six units resulted in similar physiological stability as seen in the CoolCuddle‐1 study. Despite the level of acuity seen in these infants, there was little evidence for changes in respiratory or cardiovascular measures during the CoolCuddle period. However, pain score and aEEG measures may have improved during the Cuddle. Similar to the CoolCuddle‐1 study, no evidence of adverse events was observed, and therapeutic hypothermia remained unaffected. Implementing CoolCuddle during therapeutic hypothermia and intensive care supported by the animations, rigorous and adaptable standard operating procedure with monitoring of any adverse effects may benefit parents and their infants.

## AUTHOR CONTRIBUTIONS

**Ela Chakkarapani:** Conceptualization; methodology; investigation; funding acquisition; writing – original draft; visualization; writing – review and editing; project administration; resources; supervision; validation. **Jenny Ingram:** Conceptualization; investigation; funding acquisition; writing – review and editing; project administration; resources; methodology. **Stephanie Stocks:** Project administration; resources; supervision; writing – review and editing; investigation. **Lucy Beasant:** Funding acquisition; project administration; writing – review and editing. **David Odd:** Conceptualization; investigation; validation; software; formal analysis; data curation; writing – review and editing; funding acquisition; visualization; methodology.

## FUNDING INFORMATION

This project is funded by the National Institute for Health and Care Research (NIHR) under its Research for Patient Benefit (RfPB) Programme (Grant Number NIHR203034). The views expressed are those of the author(s) and not necessarily those of the NIHR or the Department of Health and Social Care.

## CONFLICT OF INTEREST STATEMENT

The authors have no conflicts of interest to declare.

## ETHICAL APPROVAL

CoolCuddle2 received REC approval from North West‐Greater Manchester West Research Ethics Committee (22/NW/0141) and HRA approval in June 2022.
